# Persistent Activities of Extracellular Enzymes Adsorbed to Soil Minerals

**DOI:** 10.3390/microorganisms8111796

**Published:** 2020-11-16

**Authors:** Folasade K. Olagoke, Klaus Kaiser, Robert Mikutta, Karsten Kalbitz, Cordula Vogel

**Affiliations:** 1 Chair of Soil Resources and Land Use, Institute of Soil Science and Site Ecology, Technische Universität Dresden, 01062 Dresden, Germany; karsten.kalbitz@tu-dresden.de; 2 Soil Science and Soil Protection, Martin Luther University Halle-Wittenberg, 06120 Halle (Saale), Germany; klaus.kaiser@landw.uni-halle.de (K.K.); robert.mikutta@landw.uni-halle.de (R.M.)

**Keywords:** adsorption, protein, microorganisms, organic matter, specific enzyme activity, soil minerals, montmorillonite, kaolinite, goethite

## Abstract

Adsorption of extracellular enzymes to soil minerals is assumed to protect them against degradation, while modifying their activities at the same time. However, the persistence of the activity of adsorbed enzymes remains poorly understood. Therefore, we studied the persistence of cellulase and α-amylase activities after adsorption to soil amended with various amounts (+1, +5, and +10 wt.%) of three typical soil minerals, montmorillonite, kaolinite, and goethite. Soil without mineral addition (pure soil), pure minerals, and pure dissolved enzymes were used as references. Soil mineral–enzyme complexes were prepared and then incubated for 100 days; temporal changes in enzyme activities were analyzed after 0, 0.1, 1, 10, and 100 days. The specific enzyme activities (activities normalized to protein content) and their persistence (activities relative to activities at day 0) were compared to enzyme activities in solution and after sorption to the control soil. Amylase adsorption to pure minerals increased in the following order: montmorillonite > kaolinite > goethite. That of cellulase increased in the following order: goethite > montmorillonite > kaolinite. Adsorption of enzymes to soils did not increase in the same order of magnitude as the addition of reactive binding sites. Based on inverse relationships between the amount of enzyme adsorbed and the specific enzyme activity and their persistency, we showed that a limited availability of sorption sites is important for high specific activity and persistence of the enzymes. This is probably the consequence of less and weaker bonds, as compared to a high availability of sorption sites, resulting in a smaller impact on the active sites of the enzyme. Hence, we suppose that the soil mineral phase supports microorganisms in less-sorptive environments by saving energy on enzyme production, since small enzyme release could already result in sufficient activities to degrade respective target carbon substrates.

## 1. Introduction

Extracellular enzymes play a key role in soil element cycling [1,2] and are central for the microbial decomposition of soil organic matter (OM). Most OM in soil exists in close association with minerals [3,4,5], by which it is assumed to be stabilized against microbial degradation [3]. After excretion, extracellular enzymes can either exist in a free state or become adsorbed to OM and/or soil minerals. In a free state, they are likely subjected to rapid denaturation or enzymatic degradation [6]. Adsorption to soil minerals is assumed to protect enzymes against degradation [7] and to modify their activities [8,9,10]. However, the persistence of the activity of adsorbed enzymes remains poorly understood.

Adsorption of enzymes to soil increases with increasing clay contents [11]. Moreover, higher enzyme activities have been attributed to the clay-sized fraction of soils [12,13]. Schimel et al. [14] showed that the activities of several hydrolytic and oxidative enzymes persisted longer in clay-rich than in clay-poor fumigated soils. Kedi et al. [15] found higher persistence of phosphatase activity adsorbed to a sterilized Acrisol (sandy clay loam) than to a more clay-rich Vertisol (clayey), after 30 days, which was related to differences in the soil mineral composition. These results indicate that not only the content of clay-sized minerals but also the type of mineral may exert a profound influence on the amounts, as well as persistent activities of adsorbed enzymes. Divergent effects of different minerals on enzyme activities may be due to variations in adsorption sites and binding capacities [8,9,16]. So far, most studies have focused on pure clay minerals [17]; thus, knowledge on enzyme adsorption to various soil mineral types, especially within a soil matrix, is virtually non-existent. Although some of the few available studies on persistence of enzyme used natural soils, their ability to preserve enzymatic activity has been mostly interpreted based on differences in soil texture. Relating enzyme activities to the specific prevalent minerals in these soils is therefore difficult.

Depending on the soil mineral composition, interactions with enzymes may be different, and therefore persistence of enzyme activity may vary across soils. Enzymes in solution (non-adsorbed) can also be stable, as shown for different phosphatases by Carrasco et al. [18] and Kedi et al. [15]. Contrary to the usual assumption of increasing persistency when adsorbed, adsorption may even result in decreasing persistence. For instance, Carrasco et al. [18] found merely 30% of remaining activity after adsorption of alkaline phosphatase on sepiolite, a magnesium-rich phyllosilicate, in comparison to the free counterpart. These data illustrate our limited knowledge about potential effects of soil mineral–enzyme interactions, even though enzymes are likely rarely free in soils [14]. Thus, exploring the adsorption of enzymes to different soil minerals and the subsequent persistence of their activities seems of utmost importance to better understand the cycling and storage of OM in soil.

The objectives of our study were to investigate the adsorption of cellulase and α-amylase, enzymes associated with the carbon cycle, to soils amended with varying amounts of different minerals (montmorillonite, goethite, and kaolinite), and to evaluate the persistent activities (prolonged activity) of the adsorbed enzymes. Montmorillonite, goethite, and kaolinite are commonly found in soil and are thus representative for the soil minerals. We chose these three minerals because they vary in net surface charges, and, depending on the electrostatic interaction with the enzymes, they may exert varying effects on the persistence of enzymes. Additionally, enzyme–mineral studies involving goethite are less common despite its wide distribution in many soils. We selected enzymes that are involved in the breakdown of important polysaccharides, cellulose, and starch, naturally available in soils. Their application allows an improved assessment of enzymes activities in natural soils than commonly used substrates, e.g., methylumbelyferyl [19]. Cellulose, as a complex carbon compound, is highly abundant in plant litter; therefore, its enzymatic depolymerization represents a key step in the terrestrial carbon cycle. We also included starch as a less complex substrate. We hypothesized that enzyme adsorption to each mineral increases proportionally with increasing availability of sorption sites. We assumed adsorbed enzymes have reduced but longer persistence of activities than non-adsorbed (free) enzymes. Furthermore, we surmised that the degree of activity preservation is a linear function of the amount of adsorbed enzyme, which, in turn, depends on the added minerals. To address these hypotheses, we prepared enzyme–soil mineral complexes that were incubated for up to 100 days, a period considered feasible and sufficient to study the loss or persistence of enzyme activities over time [14]. The enzyme activities were measured by determining the release of glucose from cellulose and starch.

## 2. Materials and Methods

### 2.1. Sample Preparation

#### 2.1.1. Soil Pretreatment

We used a sandy soil collected from a research site located at Eberswalde-Müncheberg, Germany (52°30′55.0″ N 14°07′40.5″ E). Soil characteristics have been described in detail in Olagoke et al. [10]. The soil was selected for its low clay content (40 g kg^−1^), and it has a pH of 5.9. After collection, the soil was air-dried and sieved to <2 mm. Treatment with 10% hydrogen peroxide was used to remove OM, leaving behind residual organic carbon contents of 0.4–0.7 g kg^−1^. Thereafter, the soil was homogenized, sterilized by autoclaving three times, at 121 °C, for 1 h, and then oven-dried at 38 °C.

#### 2.1.2. Addition of Minerals

Montmorillonite, kaolinite, and goethite (α-FeOOH) were used as soil-relevant clay-sized mineral phases. Montmorillonite (montmorillonite–CERATOSIL^®^ WGD fein) was obtained from Clariant, Munich, Germany, and kaolinite (Kaolin CF70) from Caminauer Kaolinwerke, Königswartha, Germany. Goethite was prepared by slowly neutralizing 0.5 M FeCl_3_ with 1 M NaOH; the resulting precipitate was aged for 48 h at 50 °C, and then washed with ultrapure water, until the electrical conductivity was <50 µS cm^−1^, freeze dried, and finally sieved to <200 µm. The <2 µm fractions of montmorillonite and kaolinite were obtained by dispersion in deionized water and subsequent sedimentation, using Atterberg cylinders. The <2 µm fractions of the montmorillonite and kaolinite were dispersed in deionized water, before addition to soil. Goethite was dispersed in deionized water by sonication at 200 J mL^−1^ (UW 3200, Bandelin, Berlin, Germany). The sonication and addition of goethite to soil resulted in a soil/water volume of 1:5; therefore, the same ratio was used for the addition of other minerals. Each suspended mineral was added to the soil at doses of 10, 50, or 100 g kg^−1^ soil, and the suspended mixtures were stirred with a glass rod, while oven drying at 38 °C, in order to achieve a homogeneous distribution of reactive minerals. Soil without addition of minerals (pure soil), and pure minerals were used as reference materials. The pure minerals and their distribution when mixed with soil were visualized, using a scanning electron microscope (Quanta^TM^ 650 FEG, Thermo Fisher Scientific, Waltham, MA, USA). Images are presented in Figure S1. Basic characteristics of the samples, such as zeta (*ζ*) potential, specific surface area (SSA), and cation exchange capacity (CEC), were determined by standard methods and are given in Table 1. The *ζ*-potential was determined by using a Zetasizer nano (Malvern, Worcestershire, UK), and by applying the Smoluchowsky equation to electrophoretic mobility data. Measurements were carried out in water and also 0.1 M sodium acetate buffer (pH 5.5). The effective CEC was analyzed by using 0.5 M ammonium chloride (NH_4_Cl) to replace exchangeable cations [20] which were subsequently measured by inductively coupled plasma atomic emission spectroscopy (Specro Ciros CCD, Spectro Analytical Instruments, Kleve, Germany). The SSA was determined for samples degassed at 60 °C by physisorption of N_2_ gas, at −196.2 °C, using an Autosorb-1 instrument (Quantachrome, Syosset, NY, USA). The Brunauer–Emmett–Teller (N_2_-BET) method [21] was applied to 10 adsorption points in the relative pressure range 0.05–0.3.

We studied two enzymes, cellulase (EC 3.2.1) from *Aspergillus niger* (A3403) and α-amylase (EC 3.2.1.1) from *Bacillus licheniformis* (A22178) obtained from Sigma Aldrich, Munich, Germany. Cellulase and amylase activities are maximal at pH 5–6; thus, enzyme solutions were prepared in 0.1 M sodium acetate buffer (pH 5.5). Based on pretests, concentrations of 10 U mL^−1^ for amylase and 7 U mL^−1^ for cellulase were used to ensure measurable activities. The adsorption experiment was carried out according to Gianfreda and Rao [23]. Briefly, 5 mL of enzyme solution was added to 0.2 g of pure soil and minerals, as well as soil-mineral mixtures in 50 mL Eppendorf tubes. After shaking horizontally at 200 revolutions per minute (rpm), at 4 °C for 1 h, the mixture was centrifuged at 8000× *g* for 30 min, at 4 °C (8KS (143558), Sigma, Germany). The supernatants were separated, and the settled material (representing the adsorbed enzymes) was washed three times by adding 5 mL of acetate buffer, subsequent centrifuging (8000× *g* for 10 min), and decanting the supernatant. Eight replicates of adsorbed enzymes were prepared for each treatment (pure soil, pure minerals, and soil with either 10, 50, or 100 g kg^−1^ of added individual minerals). Four of them were used as substrate control, and four for glucose measurement.

The amount of adsorbed enzymes in supernatant and washing solutions was estimated by determining the protein concentration, using the Lowry method [24,25], with bovine serum albumin (A7906, Sigma Aldrich, Germany) as standard. Briefly, 100 µL of solution was added together with 100 µL of Lowry reagents in 96-well microplates and incubated for 10 min, at room temperature, in the dark. Thereafter, 100 µL of Folin’s phenol reagent (2 N diluted 10-fold in water) was added, and plates were incubated in the dark, at room temperature, for another 30 min. The absorbance was read at 750 nm, using a microplate reader (Multi-Mode Microplate Reader SynergyTM HTX, Bio-Tek Instruments, Inc., Winooski, VT, USA). Total protein in supernatant and washing solutions was subtracted from total protein in the added enzyme solution (632.7 and 945.6 µg mL^−1^ for amylase and cellulase, respectively), giving the amount of adsorbed protein. The potential release of protein from the soil and minerals was tested by treating them with enzyme-free buffer solution, as described above.

### 2.2. Determination of the Enzyme Activities

The prepared adsorption complexes were incubated at 30 °C, and then they were destructively sampled and analyzed for potential enzyme activities after 0 (immediately after preparation), 0.1, 1, 10, and 100 days. Substrate-induced activities of adsorbed and free amylase and cellulase were determined according to Deng and Popova [1]. In order to run enzyme assays at saturated substrate concentrations, we determined the substrate concentration curve for both enzymes prior to the experiment (Figure S2). Accordingly, 10% starch from corn (S4126, Sigma Aldrich Germany) was used for amylase and 2% carboxyl methyl cellulose (CMC, S3504, Sigma Aldrich, Germany) for cellulase. Both substrates were prepared in sodium acetate buffer (pH 5.5). Each 5 mL substrate was added to the soil-enzyme complexes and incubated at 30 °C. After 24 h of incubation, the mixture was centrifuged at 8000× *g* for 10 min at 4 °C, the supernatants were collected, and analyzed for reducing sugars and glucose. Soil-enzyme complexes with buffer but without substrate served as controls. In addition, pure substrate and buffer solutions were analyzed for reducing sugars and glucose. Reducing sugars were determined by using the dinitrosalicylic acid (DNS) method adapted to microplates [26], with glucose as standard. Each 25 µL of solution was added to 25 µL of the DNS reagent, in 96-well microplates. The microplates were then agitated at 450 rpm on a microtiter thermo plate shaker (PHMP-100, Grant Instruments, Cambridge, UK), at 100 °C, for 10 min. Thereafter, 250 µL of deionized water was added to each well, and the absorbance was determined at 540 nm, in a microplate reader (Multi-Mode Microplate Reader SynergyTM HTX, Bio-Tek Instruments, Inc., USA). Glucose was measured by using the GOD_POD glucose kit (Glucose oxidase (GOD) and Peroxidase (POD), NYZtech, Lisboa, Portugal). The kit was adapted to microplate use by following the manufacturer’s instruction. Each 5 µL of sample was added to 150 µL of the kit reagent, in 96-well microplates. The microplates were then shaken at 450 rpm, on a microtiter thermo plate shaker, at 40 °C, for 20 min, and the absorbance was determined at 510 nm. The specific enzyme activity of each treatment was calculated according to Gianfreda and Rao [23], as the glucose/reducing sugars content relative to the protein content (Equation (1)). The persistence of enzyme activity was expressed relative to the initial enzyme activity at day 0 for each treatment and time point. Furthermore, the supernatants of the control (soil enzyme complexes with buffer as substrate instead of cellulose or starch) were analyzed for protein (Lowry method). No protein was detected; therefore, we concluded that previously adsorbed enzymes were not released into the solution phase during incubation, i.e., the amounts of adsorbed enzymes did not change over time.

(1) $$Specific\;activity\;\left({\mu \mathrm{mol}\;\mathrm{mg}}^{-1}\min^{-1}\right)=\frac{Glucose\;content\;\left({\mu \mathrm{mol}\;L}^{-1}\min^{-1}\right)}{Protein\;content\;\left({\mathrm{mg}\;L}^{-1}\right)}$$

### 2.3. Statistical Analysis

We applied the generalized linear model (GLM), as described by Olagoke et al. [10]. The pure soil treatment (control) was used as reference for analyses of changes in specific enzyme activities over time. Then, the GLM model was fitted individually to treatments (amounts and type of minerals) and subsequently for each day and each enzyme, with the treatments as explanatory factors, and specific enzyme activities as response variables. Separation and comparison of means for the variables were done with Tukey HSD-Test at *p* < 0.05. To test for the effect of the minerals on adsorption and specific enzyme activity, correlation coefficients for the relationship between enzymes and the mineral contents were determined. All statistical data analyses were performed with R software for statistical computing, v. 3.6.2. [27].

## 3. Results

### 3.1. Enzyme Adsorption on Soil Minerals

**Figure 1:**
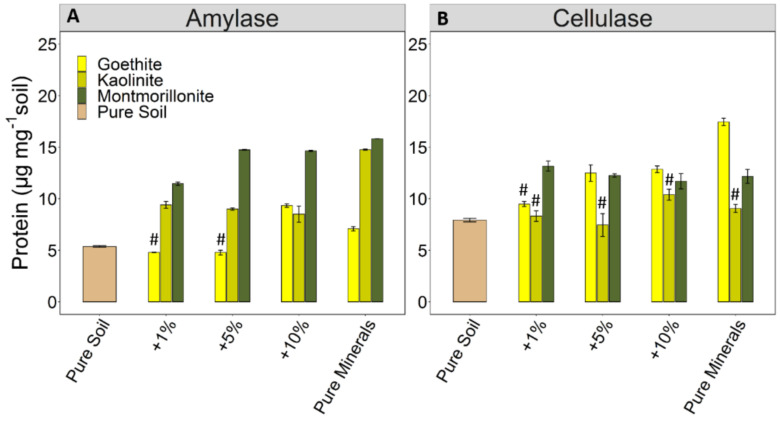
Protein content of soil, without and with added minerals, and of the pure minerals (montmorillonite, kaolinite, and goethite) after addition of amylase (**A**) and cellulase (**B**). X-axis: pure soil, soil with varying amounts of added minerals (+1%, +5%, and +10%), and pure minerals. Bars marked with # were not significantly different from the control (pure soil). Error bars represent standard error of means (*n* = 4).

Protein analyses indicate adsorption of amylase in all treatments (Figure 1A), with montmorillonite showing the highest adsorption. Nevertheless, protein contents did not increase in the same order of magnitude as addition of reactive binding sites (Figure 1A). Adsorption of amylase to goethite was little and did not exceed that of the pure soil, except for the +10% treatment. The adsorption of amylase by the pure minerals decreased in the order montmorillonite > kaolinite > goethite (Figure 1A). Cellulase was also adsorbed in all treatments (Figure 1B), but the increase in protein content with increasing mineral amount was only correlated for goethite (*r* = 0.85). The adsorption of cellulase by the pure minerals decreased in the order goethite > montmorillonite > kaolinite (Figure 1B). There was no protein release from pure soils and minerals upon addition of enzyme-free buffer.

The statistical evaluation revealed that amounts, as well as the type of mineral, explained the variation in enzyme adsorption, but the mineral type generally contributed more to the overall variance (Table 2). For amylase, the mineral type explained up to 60% of the variance, and mineral amounts about 27%. The respective numbers for cellulase were 40% and 22%.

### 3.2. Specific Activities of Adsorbed Enzymes

The two methods used to determine the enzyme activity (DNS and GOD-POD Kit) gave similar results (Figures S3–S5). Therefore, all data presented refer to glucose measurements carried out by the GOD-POD kit; the results based on reducing sugars are compiled in the supplemental material. Soil-mineral mixtures and pure minerals with no enzyme addition showed no evidence of activities throughout the studied period.

#### 3.2.1. Relationship between the Enzyme Activities and Minerals Directly after Adsorption

**Figure 2:**
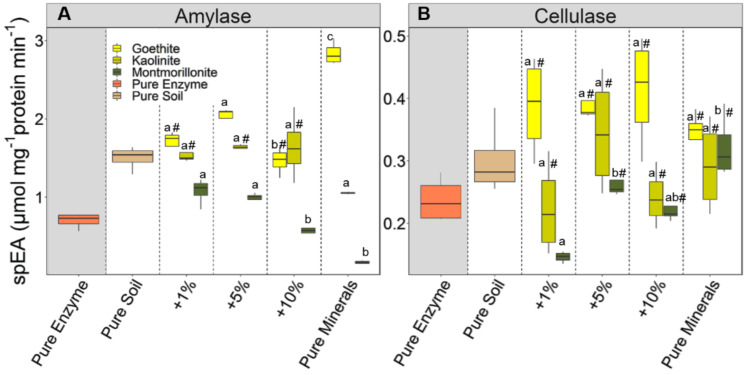
Relationship between the specific enzyme activity (spEA) of amylase (**A**) and cellulase (**B**) in response to additions of soil minerals (x-axis: pure enzyme, pure soil, soils with varying amounts of added minerals (+1%, +5%, and +10%), and pure minerals). Data present the spEA directly after addition of enzymes to the soil, with and without addition of minerals (montmorillonite, kaolinite, and goethite). Boxes (excluding the pure enzyme) marked with # are not significant (*p* < 0.05) from the control (pure soil). Boxes with same letters are not significantly different from each other. Box–whisker plots depict the median (line within boxes), first and third quartiles (upper and lower end of boxes), and non-outlier range (*n* = 4).

**Figure 3:**
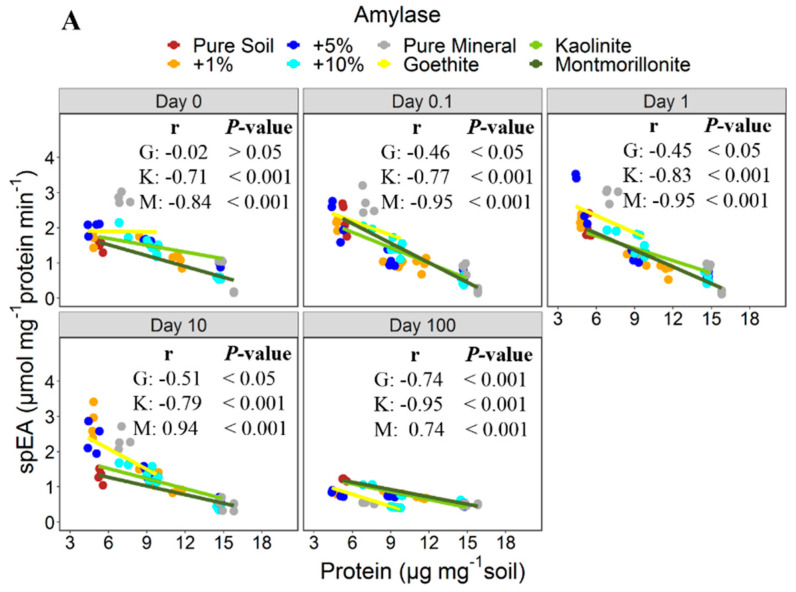

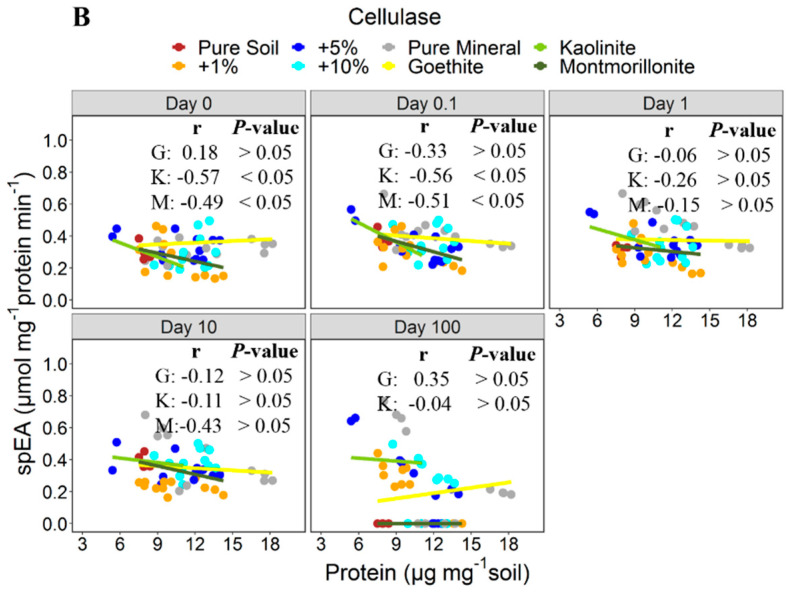
Specific enzyme activities (spEA) of amylase (**A**) and cellulase (**B**) normalized to the amount of adsorbed protein. Points represent the spEA in the pure soil, the mineral addition variants, and the pure minerals (*n* = 4). Lines indicate trends in relationships with the protein contents based on types of minerals. G = goethite, K = kaolinite, and M = montmorillonite.

The effect of minerals on the specific amylase activity varied with mineral types. It decreased with the amount of added montmorillonite (*r* = −0.79), which showed the strongest adsorption, and increased with the addition of goethite (*r* = 0.88), which adsorbed amylase little (Figure 2A). No such effect was observed for kaolinite (Figure 2A). The specific activity of adsorbed amylase was mostly higher than that of the free enzyme. Only in case of strong adsorption of amylase to montmorillonite (+10% and pure mineral) were the specific enzyme activities lower than that of the free enzyme (Figure 2A). A negative relation was found between the specific activities and the amount of adsorbed amylase (Figure 3A). For cellulase, no significant correlation was found between the specific enzyme activity and the amount of added minerals (Figure 2B) or the amount of the adsorbed enzyme (Figure 3B). Besides that, specific activity of adsorbed cellulase was either higher than or not significantly different from that of the free enzyme (Figure 2B). Noteworthy, the addition of goethite did not affect the specific activity of cellulase significantly when compared to the control, even though it adsorbed cellulase to the highest extent (Figure 2B).

#### 3.2.2. Persistence of Enzyme Activities

**Figure 4:**
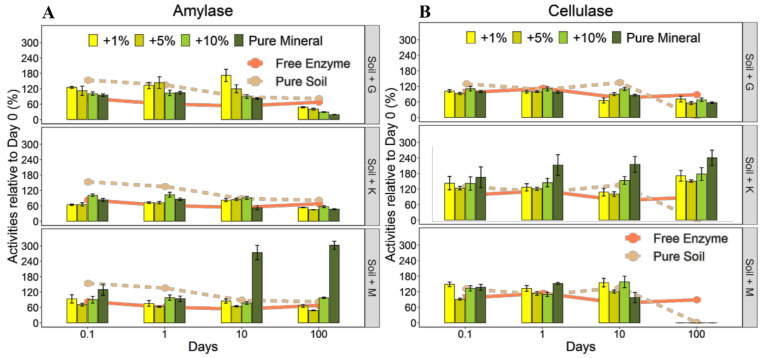
Persistence of activities of amylase (**A**) and cellulase (**B**) relative to the initial activities (at day 0) of each treatment with montmorillonite (M), kaolinite (K), and goethite (G) addition and the pure minerals. Error bars are ± standard error (SE) of means; *n* = 4. Lines represent the persistence of activities for free enzyme and in the pure soil.

**Figure 5:**
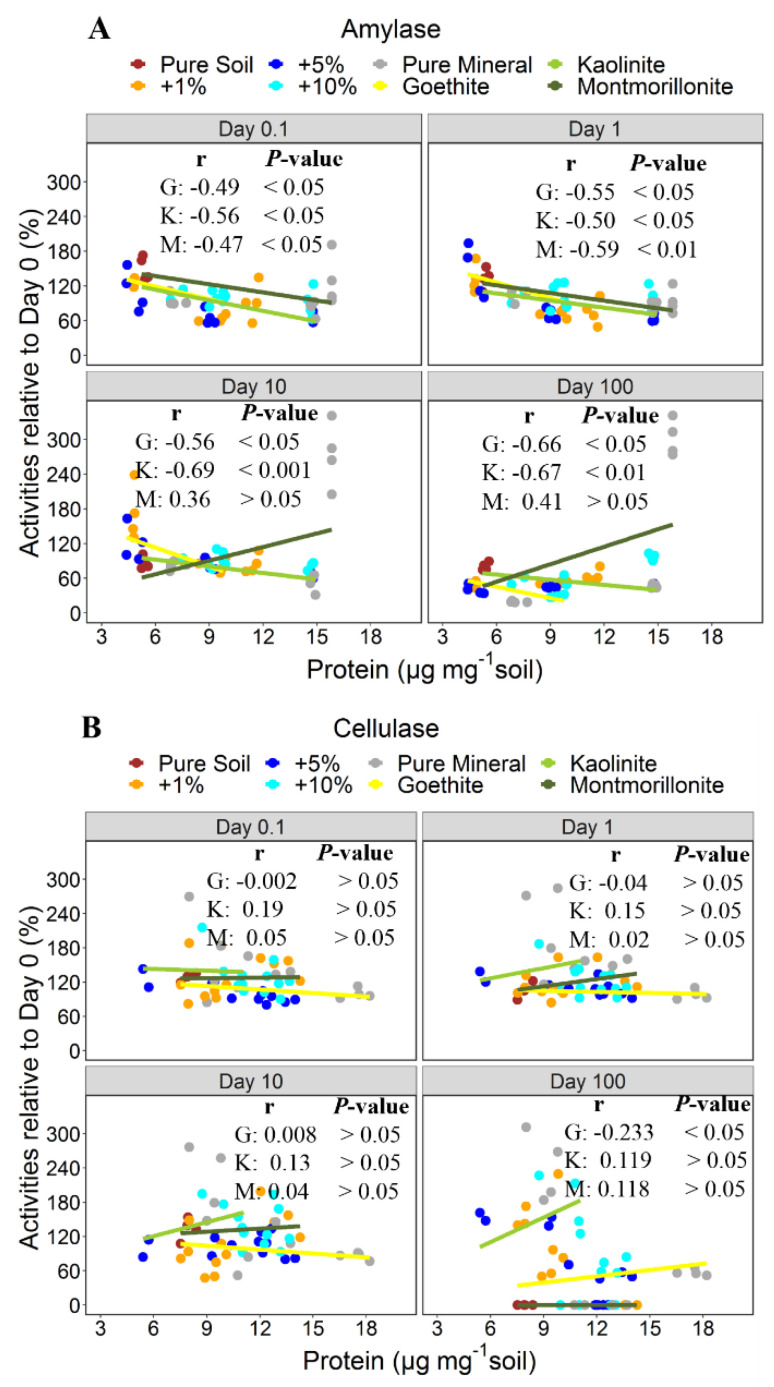
Persistence of activities of amylase (**A**) and cellulase (**B**) normalized to the amount adsorbed protein. Points represent the persistence of activities in the pure soil, the mineral additions variants, and the pure minerals (*n*=4). Lines indicate trends in relationships with the protein contents based on types of minerals. G = goethite, K = kaolinite, and M = montmorillonite.

Activities of free amylase decreased over time, but were still 67% of the initial activities after 100 days (Figure 4A). The activity of amylase adsorbed to the pure soil decreased even more during the 100 days than that of the free enzyme. Amylase bound to goethite and kaolinite variants retained activities of less than 50% and 60% by the end of the experimental period (Figure 4A). Only the 10% addition of montmorillonite and the pure montmorillonite resulted in higher activity persistence after 10 and 100 days than the free amylase (Figure 4A). Overall, montmorillonite supported the highest persistent activities and goethite the lowest over the 100-day experimental period (Figure 4A), reflecting the same order as observed for the amounts of enzymes adsorbed to the three different minerals. However, we found a significant inverse relationship between the amounts of enzymes adsorbed and the activity persistence across all minerals (Figure 5A). Only the pure montmorillonite (large adsorption of amylase) deviated from this relationship and supported a high activity at the end of the incubation (Figure 4A).

There was no significant decrease in activity of the free cellulase over time (Figure 4B). The specific activities of adsorbed cellulase were mostly higher than that of the free enzyme throughout the study period (Figure S3B). There was no evidence of an increase in the persistence of activities for adsorbed cellulase, except for kaolinite at 100 days (Figure 4B). Goethite and kaolinite resulted in an increased persistence of cellulase activity compared with the pure soil. In contrast to amylase, the amounts of adsorbed cellulase did not affect the persistence of activity (Figure 5B).

## 4. Discussion

### 4.1. Adsorption of Enzyme to the Soil Minerals

Our study shows that the adsorption to minerals decreased in the order montmorillonite > kaolinite > goethite for amylase, and in the order goethite > montmorillonite > kaolinite for cellulase. For the phyllosilicates, montmorillonite adsorbed more of both enzymes than kaolinite, but with the larger effect for amylase than cellulase (Figure 1). This is in line with previous studies on the adsorption of extracellular enzymes on pure minerals with montmorillonite retaining more enzyme than kaolinite [28,29,30] and goethite with higher sorption for β-glucosidase than montmorillonite [31].

One factor influencing enzyme adsorption to minerals is surface charge [32]. The net charge of goethite surfaces was positive at the experimental pH of 5.5, and that of the phyllosilicates, kaolinite, and montmorillonite was negative (Table 2). Accordingly, stronger electrostatic interactions would be expected to occur between negatively charged enzymes and positively charged goethite, with little attraction to negatively charged phyllosilicates. The literature data indicate that the isoelectric point of amylase ranges between 6.2 and 10 [33,34] and 3 to 4.2 for cellulase [35,36]. The pH of 5.5 in our study, therefore, should have resulted in a net positive charge for amylase, thus, favoring its adsorption to negatively charged montmorillonite and kaolinite than to goethite. However, as amylase was significantly adsorbed to pure goethite and even more to the 10% treatment (Figure 1A), the net difference in surface charge between amylase and goethite is unable to explain that observed retention. This result can likely be attributed to the aggregation of pure goethite and the distribution of goethite on the soil particles. Within the 10% treatment, goethite particles were found to be well distributed over the surfaces of soil aggregates and, thus, exposed for maximum interactions with the enzymes. By contrast, the pure goethite showed strong aggregation, indicating that parts of the goethite crystals were occluded within aggregates (Figure S1). Thus, not all sorption sites were available for enzyme adsorption. Cellulase was likely more negatively charged, which promoted its superior adsorption to positively charged goethite over that of montmorillonite and kaolinite. In addition, it is well-known that the pH at active sites of enzymes can be lower than in the bulk solution [16,29]. Such a shift in pH may influence the adsorption process by providing positively charged microsites [37,38], allowing an attraction to negatively charged surfaces of clay minerals, such as montmorillonite and kaolinite.

With the addition of different mineral amounts to the soil, a gradient in the clay-sized fraction was generated and, thus, in potential sorption sites. We expected an increasing adsorption with increasing clay-sized fraction (Figure S6). However, we could not confirm this assumption for all mineral types. We expected the strongest relationship for montmorillonite, having the highest CEC and a large SSA (Table 2). However, the used amount of amylase resulted in almost complete adsorption already when 5% of montmorillonite was added to the soil. Therefore, we did not observe any additional increase in adsorbed amylase at higher addition of montmorillonite. Conversely, the adsorption of cellulase correlated only with the added amounts of goethite (net positive charge) but was not related to added amounts of montmorillonite and kaolinite. Images obtained by scanning electron microscopy showed that the added minerals distribute well on soil aggregate surfaces at low additions, while they tend to form aggregates at high additions, similar to those of the pure minerals (Figure S1). Hence, a certain degree of homo aggregation or re-aggregation in the “high-mineral” mixtures could have masked potential sorption sites. These observations suggest that an increasing amount of clay-sized minerals control the adsorption of enzymes only if mineral and enzyme charges allow for electrostatic interactions and aggregation processes do not restrict access to sorption sites.

### 4.2. Persistence of Enzyme Activities after Adsorption to the Soil Minerals

#### 4.2.1. Comparison between Free and Adsorbed Enzymes

Our study presents a unique dataset on the temporal performance of two different enzymes, cellulase and amylase, when free or adsorbed to soil amended with different mineral types and amounts. The free enzymes used in our study persisted up to 100 days in solution, with persistent activities of about 67% and 87%, for amylase and cellulase, respectively (Figure 4). This shows that extracellular enzymes can stay active for long, even if not adsorbed to minerals surfaces. Nonetheless, we found the specific activities of the adsorbed enzymes to be higher than those of the free enzymes immediately after adsorption (Figure 2) and throughout the entire study period (Figure S3). This is in contrast to common findings of reduced activities upon enzyme adsorption to minerals [9,10,39], although most studies report potential rather than specific enzyme activities. Nevertheless, our observation aligns with the result of Giaveno et al. [40], who reported a 20% increase in potential phytase activity upon adsorption to hematite. Adsorption to minerals is known to impose changes to enzymes’ biochemical properties, such as their conformation, and thus can cause higher activities of the adsorbed, as compared to free enzymes [38,41]. Consequently, we consider it also plausible that the increased specific activity of amylase and cellulase after adsorption to minerals is related to structural changes of surface-attached enzyme molecules, potentially increasing the accessibility of active sites and/or activation of the enzymes. At the end of the study period, we could not determine a statistically consistent pattern of persistent activities due to adsorption. The persistence of activity was higher for free than adsorbed enzymes in certain instances, such as in soil amended with goethite for amylase and with montmorillonite for cellulase (Figure 4). Other treatments showed higher persistence of activities for the adsorbed than the free enzyme, e.g., in soil with montmorillonite for amylase and with kaolinite for cellulase. Kedi et al. [15] presented similar results for the activity persistence over 30 days of two phosphatases adsorbed to two sterilized soil types, a Vertisol (clayey) and an Acrisol (sandy clay loam). The authors observed, for one phosphatase, high persistence of the free enzyme, which is also in line with results by Carrasco et al. [18], reporting lower persistence of adsorbed than free phosphatase. For the other phosphatase, Kedi et al. [15] found no differences in persistent acidities in free state or when adsorbed to the Vertisol, but higher persistent activity when adsorbed to the Acrisol. Apart from degradation of enzyme over time, this loss in activity was attributed to conformational changes of the enzymes due to adsorption. In our study, while the loss in activity may be due to denaturation of the enzymes over time or inactivation, the increasing activities observed after day 0 may be attributed to activation of the enzymes in the later days, especially for amylase adsorbed on montmorillonite. Nevertheless, further insight into the rearrangement of adsorbed biomolecules and resulting activities over time is still required.

#### 4.2.2. Temporal Change of Enzyme Activities after Adsorption

The effect of enzyme adsorption to soil mineral mixtures and pure minerals on activities and their persistence varied for cellulase and amylase. The inverse relationships between specific enzyme activities and amounts of proteins being adsorbed suggest that the relative effects were most pronounced at low mineral additions and consequently when total amounts of adsorbed enzyme were small (Figure 3). It therefore seems that the activity of enzymes remains higher after sorption to a soil with a low sorption site availability, compared to soil with more abundant sorption sites. These negative relationships were generally stronger for amylase than cellulase and accompanied by the observation that the specific activity decreased with increasing mineral addition (particularly for amylase adsorption to montmorillonite; Figure 2). The presence of sufficient binding sites likely enables the enzyme to form multiple bonds to the mineral surfaces, thus increasing the probability that active sites become more oriented toward the mineral surface. Moreover, if the orientation of the enzyme active site is at the point of attachment with the minerals, this may hinder interaction with substrate, resulting in lower enzyme activities [42,43]. This was shown, using FTIR spectroscopy, by Baron et al. [42], for α-chymotrypsin. Adsorption to montmorillonite caused the amino groups at the active site of the enzyme to face the negatively charged mineral surface, thus hindering the substrate to access the active site. Increasing the adsorption capacity by minerals and using the same amounts of enzyme would mean even more of the active sites become inaccessible, as enzymes could have formed multiple bonds to mineral surfaces. On the other hand, enzyme activities seem less affected when active sites are not involved in the bonding. This situation might be the case when enzyme molecules bind to a limited number of adsorption sites and each enzyme molecule forms less bonds to the mineral surface (as likely in case of little mineral additions). However, we did not find a universal relationship between the availability of adsorption sites, the adsorption of enzymes, their specific activity, and persistence, which is applicable for different types of minerals. Nevertheless, the presumed reduction in enzyme activities due to adsorption in soils [9,10,38] may not always hold true, as the effect on each enzyme likely varies with mineral types, as well as enzymes and the respective sorption-site density provided on the mineral surfaces. Additionally, soil minerals may regulate persistence of extracellular enzymes in soil by, e.g., protection from proteolytic enzymes and direct microbial attack; however, such evidence has mostly been tested over short periods, such as 24 h [17,44].

Our results showed that pedogenic minerals vitally affect microbial processes by modifying enzyme activities. The continuous weathering during soil formation and formation of secondary minerals constantly changes the mineral composition of soils [32], and, thus, results in various interactions with soil extracellular enzymes. For example, Turner et al. [45], studying enzyme activities along the 120-kyr-old Franz Josef chronosequence in New Zealand, showed that enzyme activities were suppressed in situations where poorly crystalline minerals were abundant. Thus, the type of minerals can cause inactivation or an increased activity of enzymes. Based on our data, a minor abundance of crystalline clay-sized minerals in soil is more beneficial toward higher enzyme activities as adsorption processes under more competitive conditions might favor the formation of enzyme sorption complexes that better preserve their activity, possibly due to activation of the enzyme or less conformational changes. Although the temporal persistence of enzyme over time was mostly higher for free than adsorbed enzymes, maximum activation of extracellular enzymes by adsorption to minerals at low concentrations (i.e., low availability of sorption sites) offers an immediate benefit to microbial communities breaking down complex substrates, by saving investments in the production of extracellular enzymes. Instead, resources could be directed toward biomass production and, consequently, result in improved C- und N-use efficiency.

## 5. Conclusions

Our study shows that the adsorption of extracellular enzymes to soil minerals is largely controlled by electrostatic interactions and depended more on the type than the amount of mineral. Adsorption of enzymes to soil minerals may not always induce reduced enzyme activity, as often presumed. Changes in conformation and/or activation upon surface reactions could increase their activity. Such an increase in specific activity seems largest when only small amounts of the enzymes become adsorbed, that is, if sorption sites in soil, for example, in topsoil environments or coarse textured soils, are limited. While enzymes can be protected from degradation by adsorption to soil minerals, the induced preservation of activity may only be pronounced for enzymes not naturally stable in non-adsorbed state. Thus, adsorption to the minerals could be more beneficial for easily degradable enzymes. For soils with lower abundancies of reactive minerals, we suppose microorganisms could benefit most from enzyme adsorption to minerals by releasing only small amounts of extracellular enzymes because of the positive effect of soil minerals on their specific activity while they can invest in reproduction. In summary, adsorption-induced long- and short-term changes in activities of extracellular enzymes seem to differ across mineral types and extracellular enzymes. However, the patterns of enzyme-activity responses to sorptive interactions with minerals remain largely unresolved. Reliable prediction of possible effects would require the evaluation of binding mechanisms involved and the corresponding conformal changes of the enzymes’ chemical structure and the related changes in enzyme activities.

## Figures and Tables

**Table 1:** Basic characteristics of pure minerals and the soil used for the experiments. Abbreviations: SSA, specific surface area; CEC, cation exchange capacity. Values are shown as means ± standard error (*n* = replicates).

Soil/Minerals	ζ Potential in Water (mV) (*n* = 3)	ζ Potential in 0.1 M Sodium Acetate (mV) (*n* = 3)	SSA (m^2^ g^−1^) (*n* = 2)	Effective CEC (cmol_c_ kg^−1^) (*n* = 2)
Montmorillonite	−20.5 ± 0.1	−40.4 ± 2.1	60.7 ± 0.2	72.6 ± 0.3
Kaolinite	−35.6 ± 1.9	−43.3 ± 2.1	14.7 ± 0.1	5.3 ± 0.1
Goethite	20.0 ± 0.5	25.2 ± 0.6	84.6 ± 0.6	* 15.5 ± 1.4
Pure Soil	−19.7 ± 0.5	−23.8 ± 0.8	2.2 ± 0.2	3.7 ± 0.0

* Extracted from Mikutta et al. [22].

**Table 2:** Summary statistics of the generalized linear model (GLM) for the variation in the protein content measured for amylase and cellulase in the treatments, with or without soil mineral addition. Significance was assessed by using likelihood ratio tests. Numbers in parentheses indicate the percentage of variation explained by the respective variables.

Variables	DF	Deviance Difference *		DF	Residual Deviance ^#^		*p-*Value
		Amylase	Cellulase		Amylase	Cellulase	
NULL				51	8.40	413.66	
Amount of minerals	4	2.28 (27)	91.18 (22)	47	6.12	322.48	<0.0001
Mineral type	2	5.05 (60)	166.07 (40)	45	1.06	156.41	<0.0001

NULL: A model of response variable with no explanatory factors. * Variance explained by including a specific explanatory variable in the GLM. It represents the contribution of individual variable. ^#^ The difference between the deviance of the current model and the maximum deviance. DF = Degree of freedom.

## Supplementary Materials

The following are available as supplementary materials at https://www.mdpi.com/2076-2607/8/11/1796/s1. Figure S1: The scanning electron microscope images. Figure S2: Substrate concentration curve measured after incubation at 20 and 30 °C. Figure S3: Specific activities of adsorbed amylase and cellulase by glucose analyses obtained by measured of the glucose content. Figure S4: Specific activities of adsorbed amylase and cellulase by glucose analyses obtained by measured of the reducing sugar. Figure S5: Regression plots comparing the data obtained by glucose and reducing sugar analyses. Figure S6: Increment in the soil specific surface areas with the addition of the various amount of minerals. Table S1: The summary of generalized linear model (GLM) for the variation in specific activities of amylase and cellulase measured in soil, with or without mineral additions, over the study period.
