# Supplementary material for: Persistent Activities of Extracellular Enzymes Adsorbed to Soil Minerals
Source: Microorganisms. 2020 Nov 16;8(11):1796. doi: 10.3390/microorganisms8111796 (PMC7698205; doi:10.3390/microorganisms8111796)
Supplement: Supplementary file 1 [file microorganisms-08-01796-s001.pdf]

microorganisms

Supplementary Materials

Persistent activities of extracellular enzymes adsorbed to soil minerals

Folasade K. Olagoke[1*], Klaus Kaiser[2], Robert Mikutta[2], Karsten Kalbitz[1], Cordula Vogel[1*]

[1]Chair of Soil Resources and Land Use, Institute of Soil Science and Site Ecology, TU Dresden, Germany.
[2]Soil Science and Soil Protection, Martin Luther University Halle-Wittenberg, Germany.
[1] Correspondence: e-mail: folasade.olagoke@tu-dresden.de (F.K.O); cordula.vogel@tu-dresden.de(C.V)

Content

Visualisation of mineral and mineral distribution on soil aggregates.. 2

2 Determination of substrate concentration and enzyme activities 2

2.1 Enzyme activities measured by glucose content.. 3

2.2 Enzyme activities measured by DNS method 4

2.3 Analyses of glucose and reducing sugar results for comparability 5

3 Reference 5

Fig. S 1 The scanning electron microscope images of pure minerals (montmorillonite (M), kaolinite (K), goethite (G)) and mixed with the soil (without organic matter).. 2

Fig. S 2 Substrate concentration curve measured after incubation at 20 °C and 30 °C.......................... 3

Fig. S 3 Specific activities (spEA) of (A) amylase and (B) cellulase adsorbed to soils amended with varying amounts of either montmorillonite (M), kaolinite (K), or goethite (G)), and the pure minerals. Data obtained from analyses of glucose content. 3

Fig. S 4 Specific activities (spEA) of (A) amylase and (B) cellulase sorbed to soils amended with varying amounts of either montmorillonite (M), kaolinite (K), or goethite (G), and the pure minerals. Data obtained from analyses of the reducing sugar content.. 4

Fig. S 5 Regression plots comparing the data obtained by glucose (spEA_GLU) and reducing sugar analyses (spEA_DNS)... 5

Fig. S 6 Increment in the soil specific surface area 5

Table S 1 The summary of generalised linear model (GLM) for the variation in specific activities of amylase and cellulase measured in soil with or without mineral additions over the study................. 3

1. Visualisation of Mineral and Mineral Distribution on Soil Aggregates

After preparation of the samples, structures features and distribution of the added minerals on soil particles were analyzed by scanning electron microscope (Quanta FEG 650, Thermo Fisher Scientific, Waltham, Massachusetts, USA). The dry sample material was deposited on a double-sided adhesive carbon conductive tab and sputtered with 30nm gold. The images were conducted at 10kV beam energy and a spot size of 2.5 under high vacuum conditions.

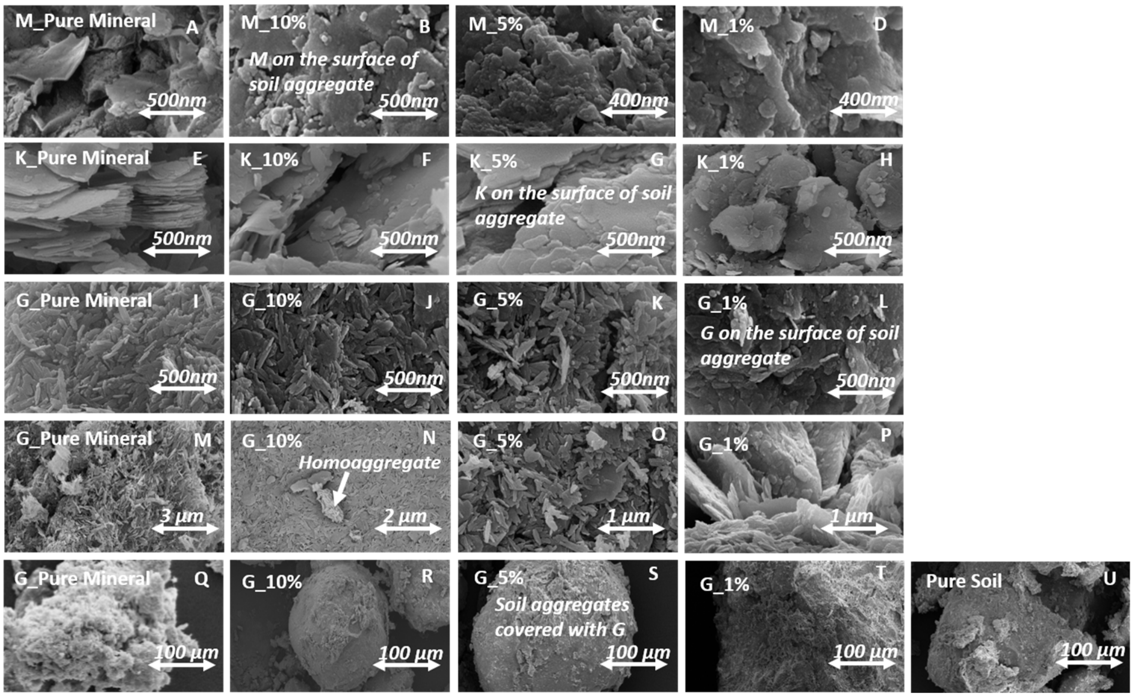

Fig*ure* S1 The scanning electron microscope images of pure minerals (montmorillonite (M), kaolinite (K), goethite (G)) and mixed with the soil (without organic matter). Percentage refers to the weight proportion of mineral. Pure soil refer to the soil without the addition of minerals

2. Determination of Substrate Concentration and Enzyme Activities

Prior to the analyses of enzyme activities, the substrate, starch and carboxyl methyl cellulose (CMC), concentration curve was determined respectively for the pure enzymes α-amylase (10 U ml^{-1}) and cellulase (7 U ml^{-1}) dissolve in 0.1 M sodium acetate buffer (pH 5.5). Six different concentration ranges between 0.1 and 20% for starch and 0.01 and 2% for CMC were used. After incubation of the enzymes with the various substrate (0.01: 1 v/v) [1] at 30°C , the glucose content of the supernatant was analysed using GOD_POD glucose kit (Glucose oxidase (GOD) and Peroxidase (POD), NYZtech, Portugal). The assay was prepared in 96 well microplate for absorbance determination at 510 nm using microplate reader (Multi-Mode Microplate Reader SynergyTM HTX, Bio-Tek Instruments, Inc., USA). Additional assay was performed at 20°C to evaluate the effect of temperature in the determination of the substrate saturation curve. The absorbance values obtained were plotted against the various concentration (Fig S2). Saturation was already attained at >5% starch for amylase and 1.5% CMC for cellulose (Fig S2). Therefore, for the determination of the enzyme

activities in our study we used 10% starch for amylase and 2% CMC for cellulase and incubation at 30°C. After the addition of substrates and incubation at 30°C, the samples were analysed for both glucose (Fig S3.) and reducing sugar (Fig S4). These results were compared using a regression analyses (Fig S5). For further analyses regarding the evaluation of the data for our objectives we run generalise linear model (GLM) (Table S1).

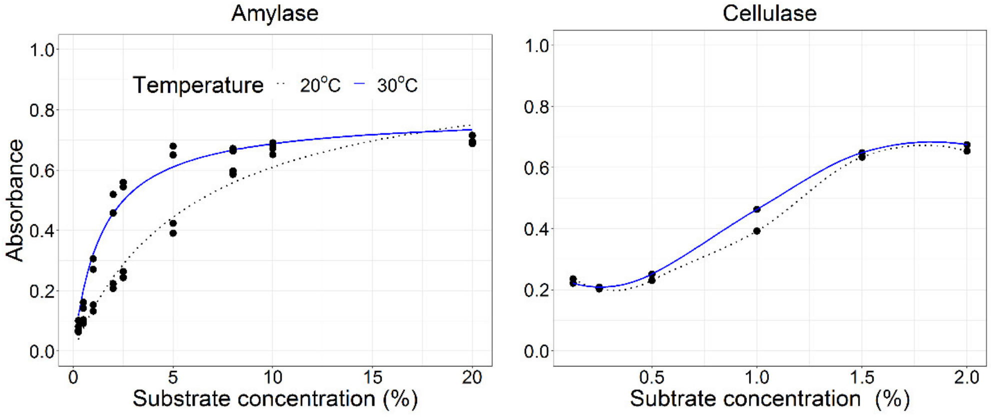

Figure S2 Substrate concentration curve measured after incubation at 20 °C and 30 °C

2.1. *Enzyme Activities Measured by Glucose Content*

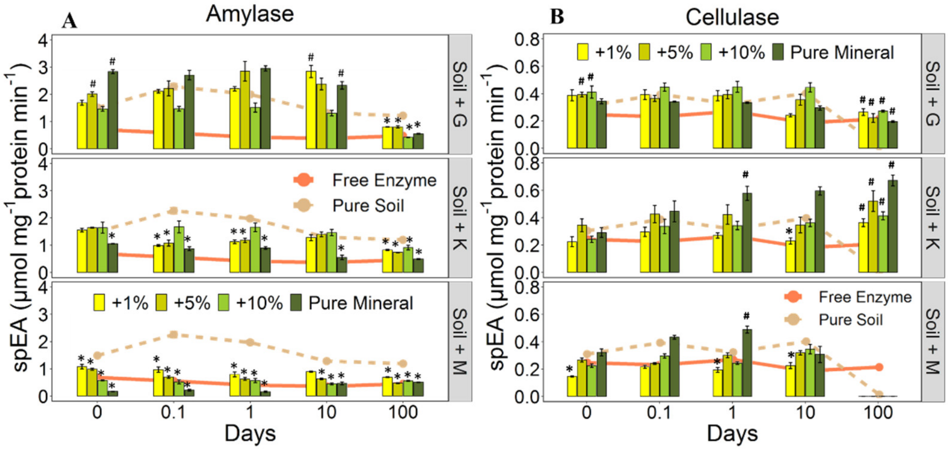

Figure S3 Specific activities (spEA) of (A) amylase and (B) cellulase adsorbed to soils amended with varying amounts of either montmorillonite (M), kaolinite (K), or goethite (G)), and the pure minerals over the experimental period of 100 days. Column marked with # (higher) and * (lower) are significant from pure soil of the same day. Data obtained from analyses of glucose content. Error bars are ± standard error (SE) of means; n=4. Lines are spEA for the free enzyme and in the pure soil.

Table S1 The summary of generalised linear model (GLM) for the variation in specific activities of amylase and cellulase measured in soil with or without mineral additions over the study period (100 days). Significance was assessed using likelihood ratio tests. Numbers in brackets indicate the percentage of variation explained by the respective variables.

Variables	Df	Deviance Difference		Df	Residual Deviance	
		Amylase	Cellulase		Amylase	Cellulase
			Day 0			

NULL				59	22.12	0.43
Mineral amount	4	0.71 (3)	0.04 (9)	55	21.4	0.38
Mineral type	2	13.66 (62)	0.18 (42)	53	7.74	0.20
Mineral amount: Mineral type	8	6.70 (30)	0.06 (14)	45	1.05	0.13
			Day 0.1			
NULL				59	14.55	0.49
Mineral amount	4	1.16 (8)	0.07 (14)	55	13.39	0.42
Mineral type	2	10.42 (72)	0.08 (16)	53	3.00	0.34
Mineral amount: Mineral type	8	1.99 (14)	0.13 (27)	45	0.98	0.21
			Day 1			
NULL				59	16.94	3.20
Mineral amount	4	0.68 (4)	1.00 (31)	55	16.26	2.19
Mineral type	2	13.65 (81)	0.66 (21)	53	2.62	1.53
Mineral amount: Mineral type	8	1.73 (10)	0.83 (26)	45	0.89	0.70
			Day 10			
NULL				59	13.39	3.10
Mineral amount	4	1.26 (9)	1.23 (40)	55	12.13	1.86
Mineral type	2	9.46 (71)	0.19 (6)	53	2.67	1.68
Mineral amount: Mineral type	8	1.93 (14)	0.78 (25)	45	0.74	0.89
			Day 100			
NULL				59	2.91	27.81
Mineral amount	4	1.66 (57)	4.39 (16)	55	1.25	23.4
Mineral type	2	0.33 (11)	22.2 (80)	53	0.92	1.21
Mineral amount: Mineral type	8	0.78 (27)	0.75 (3)	45	0.14	0.46

2.2. Enzyme Activities Measured by DNS Method

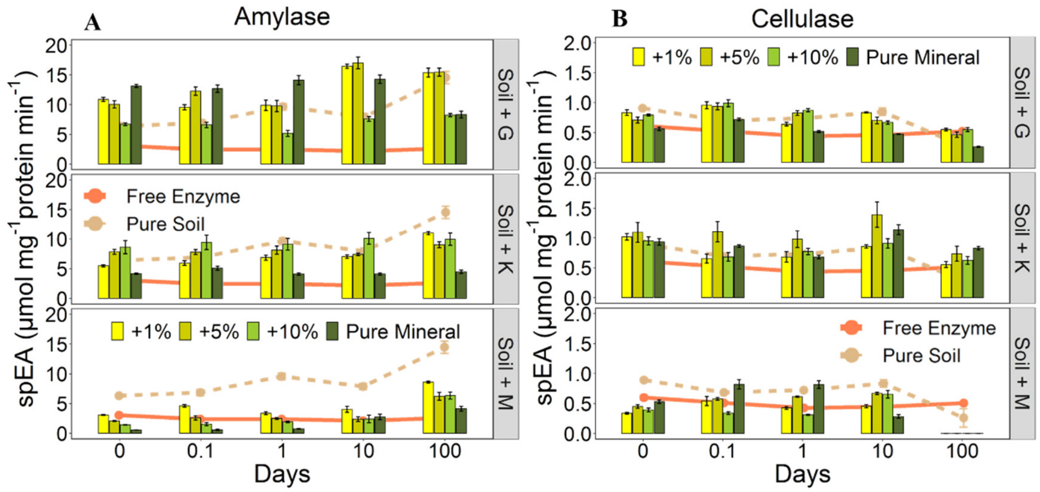

Figure S4 Specific activities (spEA) of (A) amylase and (B) cellulase sorbed to soils amended with varying amounts of either montmorillonite (M), kaolinite (K), or goethite (G), and the pure minerals over the experimental period of 100 days. Data obtained from analyses of the reducing sugar content.

Error bars are ± standard error (SE) of means; n=4. Lines are spEA for the free enzyme and in the pure soil.

2.3. Analyses of Glucose and Reducing Sugar Results for Comparability

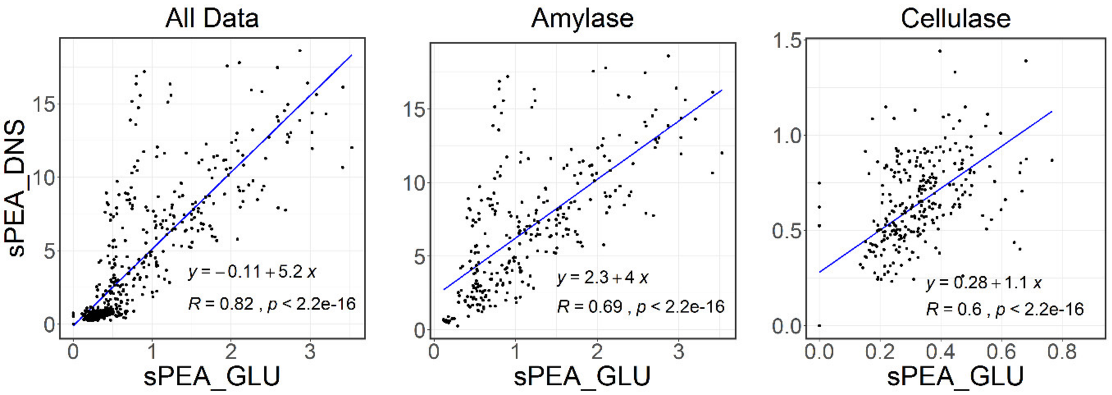

Figure S5 Regression plots comparing the data obtained by glucose (spEA_GLU) and reducing sugar analyses (spEA_DNS)

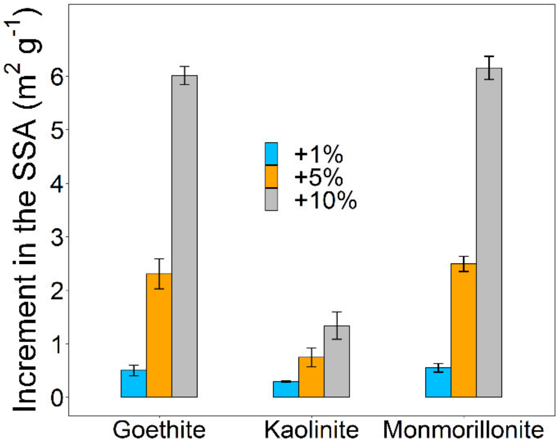

Figure S6 Increment in the soil specific surface area (SSA) with the addition of the various amount of minerals.

Reference

1. 1. Deng, S.; Popova, I. Carbohydrate Hydrolases. In *Methods of Soil Enzymology*, Dick, R.P., Ed. Soil Science Society of America: Madison, WI, 2011; http://doi.org/10.2136/sssabookser9.c9pp. 185-209.

© 2020 by the authors. Submitted for possible open access publication under the terms and conditions of the Creative Commons Attribution (CC BY) license (http://creativecommons.org/licenses/by/4.0/).
